# A Combined Linkage and Exome Sequencing Analysis for Electrocardiogram Parameters in the Erasmus Rucphen Family Study

**DOI:** 10.3389/fgene.2016.00190

**Published:** 2016-11-08

**Authors:** Claudia T. Silva, Irina V. Zorkoltseva, Najaf Amin, Ayşe Demirkan, Elisabeth M. van Leeuwen, Jan A. Kors, Marten van den Berg, Bruno H. Stricker, André G. Uitterlinden, Anatoly V. Kirichenko, Jacqueline C. M. Witteman, Rob Willemsen, Ben A. Oostra, Tatiana I. Axenovich, Cornelia M. van Duijn, Aaron Isaacs

**Affiliations:** ^1^Genetic Epidemiology Unit, Department of Epidemiology, Erasmus University Medical CenterRotterdam, Netherlands; ^2^Doctoral Program in Biomedical Sciences, Universidad del RosarioBogotá, Colombia; ^3^GENIUROS Group, Genetics and Genomics Research Center CIGGUR, School of Medicine and Health Sciences, Universidad del RosarioBogotá, Colombia; ^4^Institute of Cytology and Genetics, Siberian Branch of Russian Academy of SciencesNovosibirsk, Russia; ^5^Department of Human Genetics, Leiden University Medical CenterLeiden, Netherlands; ^6^Department of Medical Informatics, Erasmus University Medical CenterRotterdam, Netherlands; ^7^Department of Epidemiology, Erasmus University Medical CenterRotterdam, Netherlands; ^8^Department of Internal Medicine, Erasmus University Medical CenterRotterdam, Netherlands; ^9^Inspectorate of Health CareThe Hague, Netherlands; ^10^Department of Clinical Genetics, Erasmus University Medical CenterRotterdam, Netherlands; ^11^Center for Medical Systems BiologyLeiden, Netherlands

**Keywords:** genetics, epidemiology, electrocardiography, linkage, exome

## Abstract

Electrocardiogram (ECG) measurements play a key role in the diagnosis and prediction of cardiac arrhythmias and sudden cardiac death. ECG parameters, such as the PR, QRS, and QT intervals, are known to be heritable and genome-wide association studies of these phenotypes have been successful in identifying common variants; however, a large proportion of the genetic variability of these traits remains to be elucidated. The aim of this study was to discover loci potentially harboring rare variants utilizing variance component linkage analysis in 1547 individuals from a large family-based study, the Erasmus Rucphen Family Study (ERF). Linked regions were further explored using exome sequencing. Five suggestive linkage peaks were identified: two for QT interval (1q24, LOD = 2.63; 2q34, LOD = 2.05), one for QRS interval (1p35, LOD = 2.52) and two for PR interval (9p22, LOD = 2.20; 14q11, LOD = 2.29). Fine-mapping using exome sequence data identified a C > G missense variant (c.713C > G, p.Ser238Cys) in the *FCRL2* gene associated with QT (rs74608430; *P* = 2.8 × 10^-4^, minor allele frequency = 0.019). Heritability analysis demonstrated that the SNP explained 2.42% of the trait’s genetic variability in ERF (*P* = 0.02). Pathway analysis suggested that the gene is involved in cytosolic Ca^2+^ levels (*P* = 3.3 × 10^-3^) and AMPK stimulated fatty acid oxidation in muscle (*P* = 4.1 × 10^-3^). Look-ups in bioinformatics resources showed that expression of *FCRL2* is associated with *ARHGAP24* and *SETBP1* expression. This finding was not replicated in the Rotterdam study. Combining the bioinformatics information with the association and linkage analyses, *FCRL2* emerges as a strong candidate gene for QT interval.

## Introduction

The electrocardiogram (ECG) is an important tool for diagnosing, monitoring and evaluating risk in patients with cardiovascular disease (CVD; Lin et al., 2013; Pelto et al., 2013). ECG measurements, such as PR interval, QRS complex duration, and QT interval, are used for the diagnosis and prediction of cardiac arrhythmias and sudden cardiac death (SCD; Kolder et al., 2012). Myocardial depolarization and repolarization time are measured by the QT interval: the time between the onset of the QRS complex and the end of the T wave. QT shortening or prolongation has been associated with an increased risk for arrhythmias and SCD (Newton-Cheh and Shah, 2007). PR interval and QRS duration are measures of cardiac conduction time; QRS duration reflects conduction through the ventricular myocardium, while PR interval measures atrial and atrioventricular conduction from the sinoatrial node to the ventricular myocardium, primarily through the atrioventricular node (Cheng et al., 2015; Mozos and Caraba, 2015).

There are significant genetic contributions to ECG measurements; genome-wide association studies (GWAS) identified at least 71 common variants associated with their variability (Arking et al., 2006, 2014; Newton-Cheh et al., 2007, 2009; Pfeufer et al., 2009, 2010; Holm et al., 2010; Sotoodehnia et al., 2010). A number of these associations were established in loci containing genes that encode proteins with previously known roles in heart development and function, such as cardiac transcription factors; sodium, calcium, and potassium ion channels; genes with a role in myocardial electrophysiology; and others involved in the conduction of electrical impulses (Kolder et al., 2012). These include *ARHGAP24, SETBP1, LRIG1, CREBBP, MEIS1. TBX20*, and *TBX5*. Some ion channel encoding genes, such as *SCN5A, HERG, KCNE1*, and *KCNE2*, have been associated with long QT syndrome (LQTS; Tristani-Firouzi et al., 2001), atrial fibrillation (AF) and Brugada Syndrome (Hedley et al., 2011). Collectively, however, these loci explain only modest proportions of phenotypic variability; GWAS SNPs specific for each trait account for limited trait heritability (17% for QRS, 4% for QT, and 2% for PR) (Silva et al., 2015).

Genome-wide association studies generally interrogate only common variants, typically of small effect. Families, in addition to being robust against population stratification, may be enriched for less frequent variants, which can potentially be identified by linkage and fine mapping. The aim of this study, therefore, was to discover less frequent variants using linkage analysis in a large family-based study, the Erasmus Rucphen Family Study (ERF).

## Materials and Methods

### Study Population

The ERF study, which is a part of the Genetic Research in Isolated Populations (GRIP) Program, is a family-based study including over 3000 participants descendant from 22 couples that lived in the Rucphen region in the southwest Netherlands in the 19th century (Pardo et al., 2005). All descendants of those couples were invited to visit the clinical research center in the region, where they were examined in person (Aulchenko et al., 2004). Interviews at the time of blood sampling were performed by medical practitioners and included questions on a broad range of topics, including current medication use and medical history (Sayed-Tabatabaei et al., 2005). Height and weight were measured with the participant in light underclothing and body mass index (kg/m^2^) was computed. Blood pressure (BP) was measured twice on the right arm in a sitting position after at least five minutes rest, using an automated device (OMRON 711, Omron Healthcare, Bannockburn, IL, USA). The average of the two measures was used for analysis. Hypertension was defined through the use of antihypertensive medication and/or through the assessment of BP measurements according to the World Health Organization (1999) guidelines (individuals with BP ≥ 140/90 mmHg should be regarded as hypertensive). The Medical Ethics Committee of the Erasmus University Medical Center approved the ERF study protocol and all participants, or their legal representatives, provided written informed consent.

### ECG Measurement and Interpretation

Examinations included 10 s 12-lead ECG measurements, recorded with an ACTA-ECG (Esaote, Florence, Italy) with a sampling frequency of 500 Hz. Digital measurements of the ECG parameters were made using the Modular ECG Analysis System (MEANS; van Bemmel et al., 1990). Briefly, MEANS operates on multiple simultaneously recorded leads, which are transformed to a detection function that brings out the QRS complex and the other parts of the signal. MEANS determines common onsets and offsets for all 12 leads together on one representative averaged beat, with the use of template matching techniques. The measurement and diagnostic performance of MEANS have been extensively evaluated, both by the developers and by others (Willems et al., 1987, 1991; van Bemmel et al., 1990; de Bruyne et al., 1997; Eijgelsheim et al., 2009). The MEANS criteria for MI are mainly based on pathological Q waves, QR ratio, and R-wave progression (Leening et al., 2010). A cardiologist, specialized in ECG methodology, ascertained the final diagnosis of MI. QT interval was corrected for heart rate using Bazett’s formula in all analyses (Funck-Brentano and Jaillon, 1993).

### Genotyping and Statistical Analyses of the Linkage Study

Illumina’s HumanHap6k Genotyping BeadChip (*6K Illumina Linkage IV Panels*^®^) was used for genotyping for the linkage analyses. All genotyping procedures were performed according to the manufacturer’s protocols. Only markers with minor allele frequency (MAF) > 0.05 were selected for further analysis. Genotyping errors leading to Mendelian inconsistencies were detected using PedCheck (O’Connell and Weeks, 1998). Unlikely double recombination events were detected using MERLIN (Abecasis et al., 2002). All observed Mendelian errors were eliminated from the data. A total of 5250 autosomal SNPs with a call rate greater than 95% were included in the linkage analyses. All traits were adjusted for age, sex, BMI and height and inverse-normal transformation of ranks was applied before analysis. One thousand five hundred and forty-seven people with complete ECG, covariate, and genotype data were included in the initial analysis. Variance component multipoint linkage was performed using the –vc option in the MERLIN v.1.0.1 software (Gudbjartsson et al., 2000; Abecasis et al., 2002). This program calculates exact IBD sharing probabilities using the Lander-Green algorithm, requiring restriction of pedigree size. Because of this, the large single ERF pedigree with multiple loops was split into non-overlapping fragments of no more than 18 bits with the help of the PedSTR program (Kirichenko et al., 2009). Final variance component two-point linkage analysis for the identified *FCRL2* variant (rs74608430) was performed using Merlin in one large, single pedigree.

Regions of interest with LOD > 1.9 were selected for further study (Lander and Kruglyak, 1995). Borders of the linkage areas were defined as LOD score minus 2 support intervals (LOD-2 SI) around the linkage peaks. Genes within the LOD-2 SI were annotated using SCAN (SNP and CNV Annotation Database^1^).

### Exome Sequencing

Exomes for 1336 individuals from ERF were sequenced at the Center for Biomics, Department of Cell Biology, Erasmus MC, the Netherlands, using the Agilent V4 capture kit on an Illumina HiSeq2000 sequencer using the TruSeq Version 3 protocol. Mean depth base was 74.23x (median = 57x) and mean depth region was 65.26x (median = 52.87x). The sequence reads were aligned to the human genome build 19 (hg19) using BWA and the NARWHAL pipeline (Li and Durbin, 2009; Brouwer et al., 2012). The aligned reads were processed further using the IndelRealigner, MarkDuplicates, and TableRecalibration tools from the Genome Analysis Toolkit (GATK) and Picard^2^ to remove systematic biases and to recalibrate the PHRED quality scores in the alignments. Genetic variants were called using the Unified Genotyper tool of the GATK. About 1.4 million Single Nucleotide Variants (SNVs) were called and, after removing the low quality variants (QUAL < 150), we retrieved 577,703 SNVs in 1,309 individuals. Linear regression analyses, with SNVs in an additive model, were conducted on ECG measures, adjusted for age, sex, BMI, and height. To reduce the burden of multiple testing, we assessed only damaging variants in the LOD-2 SI; we found 324 such variants for QT, 52 for QRS and 61 for PR. We employed a Bonferroni correction for the number of deleterious mutations selected for each trait (QT: *P* = 1.5 × 10^-4^, QRS: *P* = 9.6 × 10^-4^, and PR: *P* = 8.2 × 10^-4^). The proportion of trait variance explained by the SNP was calculated using the Merlin software (Abecasis et al., 2002).

### Replication

We sought to replicate our findings in the Rotterdam Study (RS) cohort. The RS is an ongoing prospective cohort study conducted since 1990 in the city of Rotterdam in The Netherlands (Hofman et al., 2013). The Illumina Exome BeadChip array (“exome chip”) was developed through a large international initiative to efficiently study coding variants spanning the genome. The v1.0 array contains 247,870 variants, which were genotyped in 3,183 individuals from the RS population. Calling for this sample, and numerous others, was done centrally (in total, 62,267 samples). After rigorous quality control and exclusion of variants that were monomorphic or too rare to analyze, the final dataset consisted of 108,678 polymorphic variants in 3,163 individuals.

### Bioinformatics Analysis

To predict the functionality of genetic variants, and for comparison to BWA and NARWHAL, annotations were also performed using the dbNSFP (database of human non-synonymous SNPs and their functional predictions^3^ and Seattle^4^ databases. These databases gave functional prediction results from four different programs (PolyPhen-2, SIFT, MutationTaster, and LRT) (Chun and Fay, 2009; Adzhubei et al., 2010; Schwarz et al., 2010; Vaser et al., 2016), in addition to gene and variant annotations. Genes containing nominally significant variants (**Table 2**) were analyzed using Ingenuity Pathway Analysis (IPA; Ingenuity systems Inc, Redwood city, CA, USA). Several IPA modules were implemented: the “core analysis” was used to assess pathways, relationships, and mechanisms relevant to the dataset; the “upstream regulator analysis” was implemented to identify molecules (including microRNA and transcription factors) that may affect expression levels; and the “downstream effects analysis” was utilized to predict downstream biological processes that are increased or decreased.^5^ The GEO2R^6^ tool was used to analyse microarray-based expression data in the GEO database (GEO Accession numbers: GSE2240 and GSE41177). The Gene Network tool^7^ was used to describe co-expression networks and to assess potential functional effects of identified genes.

## Results

**Table 1** shows the characteristics of the participants included in the discovery linkage analyses and exome sequencing, as well as the exome chip replication sample. There were no significant differences between the largely overlapping linkage and exome sequence groups. The replication sample was considerably older, and was characterized by increased frequency of hypertension (and BP differences), increased PR interval and decreased QT interval compared to the discovery samples. The three ECG traits studied (the QT, QRS, and PR intervals) demonstrated only modest pair-wise correlations in the discovery dataset (**Supplementary Table 1**).

**Table 1:** Descriptive statistics of the linkage, exome sequence, and replication populations.

	Linkage Studies			Exome sequence			Exome array		
			
	ERF = 1860			ERF = 1212			Rotterdam Study = 2300		
			
	Mean (*SD*)	Minimum	Maximum	Mean (*SD*)	Minimum	Maximum	Mean (*SD*)	Minimum	Maximum
Males (n, %)	775 (42%)			488 (40%)			1012 (44%)		
Age (years)	46.4 (13.8)	16.6	85.3	47.4 (14.0)	18.2	86.1	67.9 (8.1)	55.0	101.0
BMI (kg/m^2^)	26.6 (4.6)	15.5	61.8	26.6 (4.4)	15.5	61.8	26.2 (3.6)	14.3	44.2
Height (cm)	167.4 (9.1)	143.6	196.5	166.8 (9.1)	141.0	196.5	167.4 (9.4)	137.0	198.0
Weight (kg)	75.9 (15.1)	41.9	161.0	74.2 (14.5)	42.1	161.0	73.6 (11.9)	40.0	130.8
SBP (mm Hg)	137.7 (19.1)	85.5	217.0	138.0 (19.3)	85.5	239.0	139.5 (21.7)	82.0	231.0
DBP (mm Hg)	79.6 (9.7)	54.5	120.0	79.3 (9.7)	53.5	127.5	73.8 (11.0)	36.0	129.0
Hypertension	766 (41%)			517 (43%)			1133 (49%)		
PR	152.0 (22.4)	92.0	308.0	152.8 (22.4)	96.0	308.0	165.5 (20.8)	102.0	220.0
QT	403.1 (22.4)	336.0	531.0	403.6 (22.0)	336.0	531.0	396.6 (28.5)	282.0	516.0
QRS	96.8 (9.9)	68.0	120.0	96.8 (9.9)	68.0	120.0	96.9 (10.6)	68.0	120.0


*Mean and standard deviation (SD) are given for the continuous measurements and number (percentage) is given for the categorical variables. BMI, body mass index; SBP, systolic blood pressure; DBP, diastolic blood pressure; QT, Bazett’s corrected QT interval; SD, standard deviation.*

**FIGURE 1:**
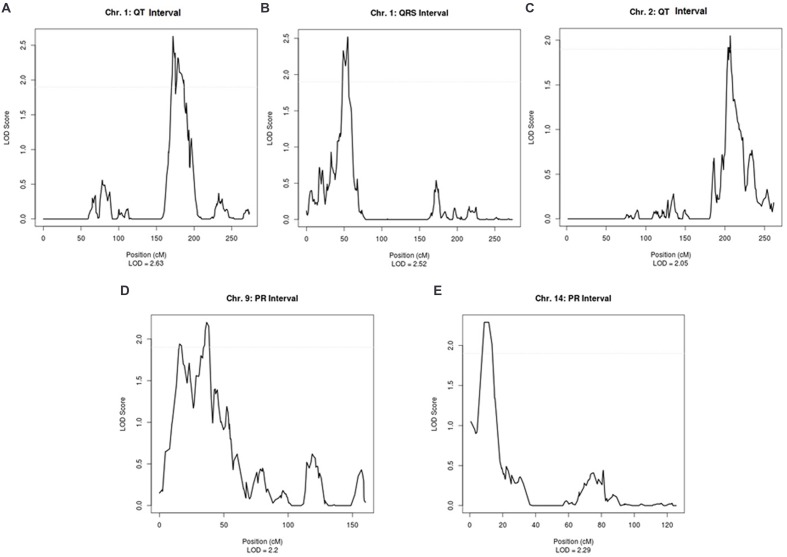
**Suggestive linkage peaks for ECG traits in ERF**.

**Supplementary Table 2** shows the linkage results for the ECG traits, which yielded a total of five regions with suggestive LOD scores (LOD > 1.9). QT was suggestively linked to two regions, on chromosome 1 (LOD = 2.63) and on chromosome 2 (LOD = 2.05). A suggestive LOD score for QRS was observed on chromosome 1 (LOD = 2.52) and, for PR, two suggestive regions were located on chromosomes 9 and 14 with LOD scores of 2.20 and 2.29, respectively (**Supplementary Table 2**). Plots of the linked regions are shown in **Figure 1**.

Our analysis of coding variants in these linked regions revealed 55,050 variants in coding regions of genes under the peaks, as described in **Table 2**. Of these mutations, 1334 had a frequency less than or equal to 5%, 437 were predicted to be damaging by at least two of the prediction software packages used, and six were nonsense variants. By linkage peak, there were 207 missense damaging mutations and two nonsense mutations on 14 and 113 missense damaging mutations and two nonsense mutations on 2q32 for QT; 51 missense mutations and one nonsense mutation on 1p36 for QRS; and 29 missense mutations on 9q21 and 31 missense mutations and one nonsense mutation on 14q12 for PR. In total, 21 variants had nominal regression *P*-values less than 0.05 (the smallest *P*-values under each linkage peak were *P* = 2.8 × 10^-4^ for QT on chromosome 1, *P* = 2.3 × 10^-2^ for QT on chromosome 2, *P* = 2.6 × 10^-2^ for QRS on chromosome 1, *P* = 1.9 × 10^-2^ for PR on chromosome 9, and *P* = 1.9 × 10^-2^ for PR on chromosome 14) without reaching the significance levels needed to account for multiple comparisons (**Supplementary Table 3**). Looking for known genes under the linkage peaks (**Supplementary Table 4**), we found two variants previously related to heart failure, *TTN* (rs72648923; *P* = 5.5 × 10^-2^, MAF = 1.4 × 10^-2^) and *HSD3B1* (*P* = 3.9 × 10^-2^ MAF = 1.1 × 10^-2^). Neither achieved statistical significance after Bonferroni correction, although both genes were marginally associated with QT. Only a single variant, a C > G (Ser > Cys) variant in *FCRL2* (rs74608430; *P* = 2.8 × 10^-4^, MAF = 1.9 × 10^-2^), approached the Bonferroni threshold for multiple-testing (*P* = 1.5 × 10^-4^). This variant, under the linkage peak on chromosome 1q23.1 for QT, is highly conserved (scorePhastCons = 0.998) and also predicted by PolyPhen-2 to be damaging (0.999). In the whole ERF population, rs74608430 explained 2.42% of the heritability of QT (reducing the LOD to 1.1; h^2^ = 0.87%; *P* = 0.02). This finding was not replicated in the RS (*P* = 0.12, β = 0.14). A sequence kernel association test analysis of the gene also failed to achieve significance in the replication sample (*P* = 0.44).

**Table 2:** Selection of the coding variants.

Trait	Locus	Variants in the coding region					Observations ≤ 5%			Predicted to be damaging			
				
		Synonymous	Missense	Stop	Splice	Total	Missense	Stop	Splice	Missense	Stop	Splice	Genes
**QT**	1	3110	5089	117	36	8353	660	0	4	207	2	0	*DENND2C, RWDD3, FCER1A, GPR25, CD1C, OMA1, LIX1L, LRRC8B, TPR, HOOK1, GTF2B, TXNIP, DDR2, CNN3, RBM15, BCL9, IVNS1ABP TNN, CEPT1, ACOT11, SARS, VAV3, TOMM40L, GABPB2, RFX5, ETV3L, APOBEC4, KIAA1614 ASPM, SPRR3, CEP350, C1orf168 COL24A1, SEMA6C, C1orf49, CACNA1S, IVL, VSIG8, EDEM3, HMCN1, TBX19, GLRX2, IFI16, PODN, INADL, MPL, HYI, CAPZA1, AMIGO1, HCN3, RTCD1, OR10J1, FLG, DMRTB1, SPTA1, HFM1, CFHR2, FCRL2, NCF2, CHIA, RBMXL1, C8A, SGIP1, FMO4, GBP1, CELSR2, ODF2L, PEAR1, FCRL1, SLC44A5, UROD, MOBKL2C, LRRC7, LRRC8C, IPO9, PRPF38B, MSH4, KIFAP3, LAMC2, PAQR6, ZNF687, MIER1, SMG7, TMEM61, ALX3, FAM189B, PDE4DIP, ATPAF1, C1orf50, PRRC2C, ZNF281, IGSF3, CRCT1, UQCRH, SLC27A3, NPHS2, PKLR, ATP1A4, TMEM125, TNR, OVGP1, SHCBP1L, UHMK1, B4GALT2 RNF220, PIAS3, KIF2C, TARS2, TMEM59, PIGK, CMPK1, PIK3R3, METTL11B CITED4, EFCAB7, TTF2, AXDND1, DDX20, IGSF9, LEPRE1, ADAMTSL4 WDR77, GNAT2, GPSM2, PPM1J, ABCA4, EXTL2, AP4B1, HIVEP3, UBQLN4, POLR3C, NEGR1, TBX15, GBP6, KIAA1324 DPYD, F5, GJA5, CYP4A22, HENMT1, MRPL37, TDRD5, ZBTB7B, SPATA6, FCRLB, ABL2, ZFYVE9, LAMC1, RHBG, DUSP12, ZYG11A, WDR3, FAAH, C1orf106 HSD3B1, CTSS, TRIM45, ALG6, ACP6, PRUNE, TRIM46, AGL, MAGI3, C1orf27, AL359075.1 SLC5A9, EBNA1BP2, COL11A1, FGGY, AMPD1, FAM63A, GLT25D2, DMRTA2, EVI5, DPT, OR6P1*
	2	1662	2444	40	17	4165	328	0	2	113	2	0	*CRYGA, TTN, ARMC9, GTF3C3, ADAM23, ZFAND2B, PER2, COL6A3, TNS1, PAX3, HDAC4, OBSL1, CAPN10, IGFBP5, TMEM198, ESPNL, SPAG16, COL4A3, ANKAR, NEUROD1, NOP58, DNAH7, IQCA1, CCDC141, KIF1A, CASP10, SSFA2, CRYGC, ECEL1, AP1S3, COL5A2, NDUFS1, ATF2, STK36, UNC80, ABCB6, KIAA1486, ANKMY1, C2orf67, PLEKHM3, CNPPD1, ALPP, EFHD1, ZSWIM2, C2orf62, AQP12B, WIPF1, PDE11A, GLB1L, CCDC150, DGKD, SERPINE2, ABCA12, ITGAV, IDH1, SPHKAP, FN1, CDK15, GPR35, WNT10A, CYP27A1, ACSL3, ANKZF1, DNAJC10, FBXO36, STK16, MYO1B, KLHL30, PIKFYVE, DES, ASNSD1*
**QRS**	1	1057	1446	25	17	2546	152	1	4	51	1	0	*OTUD3, PHC2, SYF2, DHDDS, EPB41, NBPF3, ZBTB40, COL16A1, RAP1GAP, C1orf38, EPHA10, MACF1, PADI4, LDLRAP1, RCC2, AK2, SEPN1, TMCO2, HSPG2, MAP3K6, TMCO4, CCDC28B, TMEM234 GRHL3, ALDH4A1, GJB4, MAN1C1, SERINC2, E2F2, MUL1, PHACTR4, MYOM3, SRRM1, RLF, TINAGL1, KIAA0319L, C1orf94, C1orf63, UBXN11, USP48*
**PR**	9	375	656	8	5	1053	96	0	0	29	0	0	*DENND4C, CA9, FRMPD1, PLIN2, CCIN, IFT74, UBAP1, IFNA10, RECK, UNC13B, GRHPR, KIAA1045, FREM1, OR2S2, IFNA14, FAM154A, KIAA1797, RGP1, ALDH1B1, NOL6, (GALT; GALT; RP11-195F19.29), PTPLAD2, DDX58*
	14	440	792	24	6	1276	86	0	1	31	1	0	*HEATR5A, RABGGTA, LRRC16B, RBM23, CMA1, SUPT16H, MMP14, PARP2, CEBPE, OR4K1, PRKD1, LRRC16B, MYH6, PSMB11, HEATR5A, LRP10, LRRC16B, TTC5, OR10G3, OR4N5, MYH6, TEP1, SDR39U1, TEP1, SLC7A7, LRP10, TEP1, ADCY4, (AL163636.2;AL163636.2;AL163636.2;RNASE4;RNASE4; RNASE4), PCK2, ARHGEF40, KLHL33*


*Overview of variants predicted to be damaging for each trait with a frequency or less than 5%.*

Not much is known about the function of *FCRL2*. Among the functions predicted by Gene Network are the regulation of cytosolic Ca^2+^ levels (*P* = 3.3 × 10^-3^) and AMPK stimulated fatty acid oxidation in muscle (*P* = 4.1 × 10^-3^). In the GEO database, *FCRL2* expression was higher in AF (Barth et al., 2005; Yeh et al., 2013). **Supplementary Figure 1A** shows the genes co-expressed with *FCRL2*, according to Gene Network. Two genes that have been associated with ECG outcomes by GWAS emerge: *ARHGAP24*, associated with PR, and *SETBP1*, associated with QRS (Holm et al., 2010; Pfeufer et al., 2010; Sotoodehnia et al., 2010). In the chromosome 1 region linked to QT, looking for co-expression, we found correlations between *DMRTA2. CEP350*, and *MPL* with genes previously associated with ECG traits: *DMRTA2* is co-expressed with *LRIG1*, a QRS associated gene (**Supplementary Figure 1B**); *MPL* is in a module with *MEIS1*, associated with PR (**Supplementary Figure 1C**); and *CEP350* interacts with *CREBBP*, associated with QT (**Supplementary Figure 1D**). These three genes are not in linkage disequilibrium with each other. At the chromosome 2q34 locus linked with QT, a heart failure gene, *TTN*, was under the linkage peak. According to Gene Network analysis, expression of *TTN* is related to expression of three previously known QT genes (*ATP1B, TCEA3*, and *PLN*) and two QRS and PR associated genes (*TBX20* and *TBX5*) (**Supplementary Figure 1E**) (Holm et al., 2010; Pfeufer et al., 2010; Sotoodehnia et al., 2010; Arking et al., 2014). Additionally, *SPHKAP*, on chromosome 2 under the QT linkage peak, is co-expressed with *TBX5* (**Supplementary Figure 1F**).

## Discussion

Linkage analysis is an important tool for the identification of genomic regions influencing trait variability. The role of *TPM1* mutations with sudden death is a clear example of a locus discovered by linkage analysis (Ott et al., 2011; Mango et al., 2016). The advantages of family studies include control of heterogeneity and population stratification (Reich et al., 2005; Ott et al., 2011). We performed a linkage study on ECG measurements and identified five suggestive regions (1p35.1, 1q24.2, 2q34, 9p22.2, 14q11.2). Rare variant analysis in these regions uncovered two genes related to heart failure, *TTN* (*P* = 5.5 × 10^-2^) and *HSD3B1* (*P* = 3.9 × 10^-2^) and one gene with unknown cardiac function *FCRL2* (*P* = 2.8 × 10^-4^). None of them reaches statistical significance level after correction for multiple comparisons.

This study was conducted in a large, well-characterized family-based cohort, ascertained on the basis of genealogy and not phenotype. Multiple levels of genetic data, including a linkage panel and exome sequence data, provided a powerful dataset for identifying variants that may not be easily discovered with GWAS. Unfortunately, exome data was not available in the whole cohort, which could limit our ability to identify causal variants. Additionally, the sequence data did not include extra-genic or intronic variants that may be responsible for the observed linkage peaks.

Our analysis of rare coding variants in these linkage regions revealed 55,050 variants in coding regions. One thousand three hundred and thirty-four of these mutations had a frequency less than or equal to 5% and 437 were predicted to be damaging; none reached the significance threshold accounting for multiple comparisons. These variants spanned genes, including *TTN* and *HSD3B1*, which have been previously related to CVDs. *HSD3B*, a gene on chromosome 1 (1p13.1), has two isoforms (HSD3B1 and HSD3B2) that were found to be associated with an increase in plasma aldosterone (Shimodaira et al., 2010). Changes in circulating aldosterone levels can modulate BP and hypertrophy (HT). A genome wide linkage analysis revealed that *HSD3B1* is a locus for BP variation (Shimodaira et al., 2010).

Another interesting gene covered by these variants was *TTN*; this gene encodes a sarcomeric protein named Titin, with a crucial role in sarcomeric structural integrity and muscle elasticity. Mutations in *TTN* have been shown to cause heart failure in humans. Additionally, mouse models with *TTN* mutations exhibit weak heart contractility and heart failure (Gerull et al., 2002; Xu et al., 2002; May et al., 2004) and hearts of mutant embryos displayed weak spontaneous contraction (May et al., 2004). Additionally, the *TTN* network includes three QT associated genes, *ATP1B, TCEA3*, and *PLN. TBX320*, a QRS associated gene; and *TBX5* (a QRS and QT associated gene).

We also identified a less frequent C > G missense variant (rs74608430) in the *FCRL2* gene under the linkage peak on chromosome 1p23.1. This variant explains 2.42% (h^2^ = 0.87%, *P* = 0.02) of the total genetic variance of QT (h^2^ = 36%) in the ERF population. *FCRL2* has not been previously described with respect to cardiac function. Bioinformatics resources, however, showed that *FCRL2* expression is associated with *ARHGAP24* and *SETBP1* expression, two genes implicated in ECG variability by GWAS. This suggests that *FCRL2* may be relevant for heart function. *FCRL2* is expressed mostly in liver, heart, testis and kidney^8^. Gene Network predicts that it may be relevant for cytosolic Ca^2+^ levels and AMPK stimulated fatty acid oxidation in muscle. These are plausible pathways for QT function. This finding for rs74608430, however, was not replicated in the RS, in which the MAF was 2.9 × 10^-2^. The absence of replication could be related to environmental differences influencing complex gene-environment interactions between these two study groups (Teo et al., 2009). Another plausible explanation is that, due to longer stretches of linkage disequilibrium in the family-based ERF sample, rs74608430 is tagging another variant in ERF and this is not the case in the general population.

Further, Ingenuity analysis revealed that *FCRL2* is correlated with some microRNAs (such as miR-1263, miR337-5p, miR-4699-3p, miR518e-3p, miR-507, miR3689a-5p, miR-507, miR-3622a-5p, miR-450b-5p, miR-4720-3p, and miR-1253). Among these, miR-337-5p is known to be differentially expressed in patients with valvular heart disease and patients with chronic AF (Cooley et al., 2012). This is consistent with the GEO database at NCBI^9^, which suggests that *FCRL2* is upregulated in patients with AF and dilated cardiomyopathy. In summary, the bioinformatics data available for this gene supports the hypothesis that *FCRL2* may be involved in heart function, and, specifically, related to ECG variability.

Additional interesting genes have been uncovered under the linkage peaks. First, the PR linkage peak on chromosome 14 contains damaging variants in the alpha and beta subunits of cardiac myosin *MYH6* and *MYH7.* Previous studies showed that genetic variants in these two genes have been found in hypertrophic cardiomyopathy (Anan et al., 1994; Lankford et al., 1995; Rayment et al., 1995; Niimura et al., 2002; Carniel et al., 2005), dilated cardiomyopathy (Kamisago et al., 2000; Carniel et al., 2005) and atrial septal defect (Ching et al., 2005). Second, we found *TNNT2* under the linkage peak on chromosome 1 for QT, which harbors known mutations underlying hypertrophic cardiomyopathy (Thierfelder et al., 1994) and familial dilated cardiomyopathy (Kamisago et al., 2000).

No explanatory variants were found for the other loci, for which there are a number of potential explanations. Linkage peaks are not precise in highlighting the location of the causal variant; even the region of interest cannot be easily pinpointed. Additionally, we did not take into account alternative forms of genetic variation, such as structural and copy number variations (CNVs) or repeats in the linkage regions. Lastly, causal rare variants may be located outside the coding sequence, which we did not include in our sequencing analyses.

## Conclusion

Although the combination of linkage and exome sequencing did not lead to the identification of a causal variant, suggestive linkage regions contain a number of plausible candidate genes, including *FCRL2. TTN, MYH6, MYH7, TNNT2*, and *HSD321*. Further analysis will need to be performed to demonstrate the involvement of these proteins in ECG measurements. We could not explain these with exonic sequence variants, so they will require more extensive follow-up, but provide potentially important indicators of the location of variation influencing ECG.

## Author Contributions

CS: Formal analysis, writing – original draft preparation; IZ: Formal analysis; NA: Formal analysis; AD: Formal analysis; EvL: Formal analysis; JK: Formal analysis, investigation, software; MvB: Formal analysis; BS: Investigation, resources; AU: Investigation, resources; AK: Formal analysis, software; JW: Investigation, resources; RW: Writing – original draft preparation, supervision; BO: Investigation, resources; TA: Formal analysis, supervision; CvD: Conceptualization, formal analysis, investigation, resources, writing – original draft preparation, supervision; AI: Conceptualization, formal analysis, writing – original draft preparation, supervision.

## Conflict of Interest Statement

The authors declare that the research was conducted in the absence of any commercial or financial relationships that could be construed as a potential conflict of interest.

## Footnotes

1 http://www.scandb.org

2 http://picard.sourceforge.net

3 http://varianttools.sourceforge.net/Annotation/DbNSFP

4 http://snp.gs.washington.edu/SeattleSeqAnnotation/

5 https://www.ingenuity.com/wp-content/themes/ingenuity-qiagen/pdf/ipa/ipa_datasheet.pdf

6 http://www.ncbi.nlm.nih.gov/geo/geo2r/

7 http://genenetwork.nl:8080/GeneNetwork

8 http://www.bioinfo.mochsl.org.br/miriad/gene/FCRL2/

9 http://www.ncbi.nlm.nih.gov/geo/
