# Supplementary material for: A Combined Linkage and Exome Sequencing Analysis for Electrocardiogram Parameters in the Erasmus Rucphen Family Study
Source: Front Genet. 2016 Nov 8;7:190. doi: 10.3389/fgene.2016.00190 (PMC5099142; doi:10.3389/fgene.2016.00190)
Supplement: Supplementary file 1 [file Data_Sheet_1.docx]

**Supplementary Table 1: Pearson’s correlations between ECG measures**

|  | Unadjusted | Adjusted |
| --- | --- | --- |
| QT - QRS | 0.22 | 0.16 |
| PR - QRS | 0.14 | 0.02 |
| PR - QT | 0.23 | 0.18 |

Adjusted model included age, sex, body-mass index, and height.

**Supplementary Table 2: Suggestive linkage results (LOD > 1.9) for the ECG traits measures**

|  |  |  |  |  |  |
| --- | --- | --- | --- | --- | --- |
|  | N | Chr. | SNP | Position (cM) | LOD_MAX_ |
| QT | 1547 | 1  2 | rs969310  rs1396828 | 172.03  206.59 | 2.63  2.05 |
| QRS |  | 1 | rs536766 | 56.67 | 2.52 |
| PR |  | 9  14 | rs960232  rs12586866 | 36.66  8.76 | 2.20  2.29 |

N: sample size; Chr.: chromosome; LOD_MAX_: LOD score at SNP.

**Supplementary Table 3: Selected damaging variants (*P* < 0.05) in the coding regions of genes under the linkage peaks**

| Trait | chr:pos | All | Freq | # | n | β | SE | ***P*** | *P*_HWE_ | rsID | **Gene** |
| --- | --- | --- | --- | --- | --- | --- | --- | --- | --- | --- | --- |
| **QT** | 1:179959750 | A/**G** | 1.64X10^-2^ | 34 | 1036 | 0.36 | 0.18 | 4.60X10^-2^ | 1.00 | rs140306241 | *CEP350* |
| **QT** | 1:155148520 | **A**/G | 2.31X10^-2^ | 48 | 1037 | 0.30 | 0.15 | 4.00X10^-2^ | 1.00 | rs80254867 | *TRIM46* |
| **QT** | 1:120056820 | A/**G** | 1.11X10^-2^ | 23 | 1037 | 0.45 | 0.22 | 3.88X10^-2^ | 1.00 | - | *HSD3B1* |
| **QRS** | 1:26620806 | **C**/T | 1.69X10^-2^ | 35 | 1038 | 0.36 | 0.17 | 3.88X10^-2^ | 1.00 | rs56039743 | *UBXN11* |
| **QT** | 1:43804340 | **A**/G | 1.35X10^-2^ | 28 | 1037 | 0.39 | 0.19 | 3.79X10^-2^ | 1.00 | rs12731981 | *MPL* |
| **QRS** | 1:26784304 | **A**/G | 1.11X10^-2^ | 23 | 1038 | 0.47 | 0.21 | 2.59X10^-2^ | 1.00 | - | *DHDDS* |
| **QT** | 1:50884536 | C/**T** | 1.59X10^-2^ | 33 | 1035 | -0.41 | 0.18 | 2.33X10^-2^ | 1.00 | rs200890998 | *DMRTA2* |
| **QT** | 1:183617693 | **A**/G | 2.17X10^-2^ | 45 | 1037 | -0.35 | 0.15 | 2.05X10^-2^ | 1.00 | rs16861394 | *APOBEC4* |
| **QT** | 1:159912899 | C/**T** | 3.29X10^-2^ | 68 | 1032 | 0.29 | 0.12 | 1.56X10^-2^ | 0.09 | rs3747617 | *IGSF9* |
| **QT** | 1:150972959 | A/**T** | 2.51X10^-2^ | 52 | 1037 | 0.36 | 0.14 | 1.17X10^-2^ | 0.48 | rs140386498 | *FAM63A* |
| **QT** | 1:117142844 | C/**T** | 1.54X10^-2^ | 32 | 1037 | -0.44 | 0.17 | 1.07X10^-2^ | 1.00 | - | *IGSF3* |
| **QT** | 1:157738374 | **C**/G | 1.88X10^-2^ | 39 | 1037 | -0.58 | 0.16 | 2.84X10^-4^ | 1.00 | rs74608430 | *FCRL2* |
| **QT** | 2:202074207 | A/**G** | 2.46X10^-2^ | 51 | 1037 | -0.28 | 0.14 | 4.84X10^-2^ | 1.00 | rs17860405 | *CASP10* |
| **QT** | 2:220113159 | C/**T** | 1.83X10^-2^ | 38 | 1037 | -0.34 | 0.17 | 4.38X10^-2^ | 1.00 | rs17849638 | *STK16* |
| **QT** | 2:228846564 | C/**G** | 1.16X10^-2^ | 24 | 1037 | 0.45 | 0.21 | 3.04X10^-2^ | 1.00 | rs61755300 | *SPHKAP* |
| **QT** | 2:219678877 | C/**T** | 1.59X10^-2^ | 33 | 1037 | -0.40 | 0.18 | 2.29X10^-2^ | 1.00 | rs41272687 | *CYP27A1* |
| **PR** | 9:19336290 | C/**T** | 9.17X10^-3^ | 22 | 1199 | -0.44 | 0.21 | 4.08X10^-2^ | 0.09 | - | *DENND4C* |
| **PR** | 9:34241528 | **A**/C | 1.37X10^-2^ | 33 | 1202 | 0.36 | 0.18 | 3.80X10^-2^ | 1.00 | - | *UBAP1* |
| **PR** | 9:35398605 | **A**/G | 2.66X10^-2^ | 64 | 1202 | -0.29 | 0.13 | 1.88X10^-2^ | 0.20 | rs41315995 | *UNC13B* |
| **PR** | 14:24909629 | **A**/C | 1.96X10^-2^ | 47 | 1200 | -0.30 | 0.15 | 4.21X10^-2^ | 1.00 | rs11538256 | *SDR39U1* |
| **PR** | 14:23587838 | G/**T** | 1.04X10^-2^ | 25 | 1201 | 0.47 | 0.20 | 1.93X10^-2^ | 1.00 | rs141903485 | *CEBPE* |

chr:pos: chromosome:position, All: Alleles (**bold**: major allele), #: number of alleles observed, n: sample size, β: effect size estimate, SE: standard error of β, *P*: *P*-value, *P*_HWE_: Hardy-Weinberg *P*-value.

**Supplementary Table 4A: Genes in the LOD-2 SI for the chromosome 1 QT linkage peak**

| **Symbol** | **Full name** |
| --- | --- |
| *ABCA4* | ATP-binding cassette, sub-family A (ABC1), member |
| *ABL2* | v-abl Abelson murine leukemia viral oncogene homolog 2 (arg, Abelson-related gene |
| *ACOT11* | acyl-CoA thioesterase 11 |
| *ACP6* | acid phosphatase 6, lysophosphatidic |
| *ADAMTSL4* | ADAMTS-like 4 |
| *AGL* | amylo-1, 6-glucosidase, 4-alpha-glucanotransferase |
| *AL359075.1* | Protein transport protein Sec16B |
| *ALG6* | asparagine-linked glycosylation 6, alpha-1,3-glucosyltransferase homolog (S. cerevisiae) |
| *ALX3* | ALX homeobox 3 |
| *AMIGO1* | adhesion molecule with Ig-like domain 1 |
| *AMPD1* | adenosine monophosphate deaminase 1 (isoform M) |
| *AP4B1* | adaptor-related protein complex 4, beta 1 subunit |
| *APOBEC4* | apolipoprotein B mRNA editing enzyme, catalytic polypeptide-like 4 (putative) |
| *ATP1A4* | asp (abnormal spindle) homolog, microcephaly associated (Drosophila) |
| *ATPAF1* | ATPase, Na+/K+ transporting, alpha 4 polypeptide |
| *AXDND1* | ATP synthase mitochondrial F1 complex assembly factor 1 |
| *B4GALT2* | UDP-Gal:betaGlcNAc beta 1,4- galactosyltransferase, polypeptide 2 |
| *BCL9* | B-cell CLL/lymphoma 9 |
| *C1orf106* | chromosome 1 open reading frame 106 |
| *C1orf168* | chromosome 1 open reading frame 168 |
| *C1orf27* | chromosome 1 open reading frame 27 |
| *C1orf49* | chromosome 1 open reading frame 49 |
| *C1orf50* | chromosome 1 open reading frame 50 |
| *C8A* | complement component 8, alpha polypeptide |
| *CACNA1S* | calcium channel, voltage-dependent, L type, alpha 1S subunit |
| *CAPZA1* | capping protein (actin filament) muscle Z-line, alpha 1 |
| *CD1C* | CD1c molecule |
| *CELSR2* | cadherin, EGF LAG seven-pass G-type receptor 2 (flamingo homolog, Drosophila) |
| *CEP350* | centrosomal protein 350kDa |
| *CEPT1* | choline/ethanolamine phosphotransferase 1 |
| *CFHR2* | complement factor H-related 2 |
| *CHIA* | chitinase, acidic |
| *CMPK1* | cytidine monophosphate (UMP-CMP) kinase 1, cytosolic |
| *CNN3* | calponin 3, acidic |
| *COL11A1* | collagen, type XI, alpha 1 |
| *CRCT1* | cysteine-rich C-terminal 1 |
| *CTSS* | cathepsin S |
| *CYP4A22* | cytochrome P450, family 4, subfamily A, polypeptide 22 |
| *DDR2* | discoidin domain receptor tyrosine kinase 2 |
| *DDX20* | DEAD (Asp-Glu-Ala-Asp) box polypeptide 20 |
| *DENND2C* | DENN/MADD domain containing 2C |
| *DMRTA2* | DMRT-like family A2 |
| *DMRTB1* | DMRT-like family B with proline-rich C-terminal, 1 |
| *DPT* | Dermatopontin |
| *DUSP12* | dual specificity phosphatase 12 |
| *EBNA1BP2* | EBNA1 binding protein 2 |
| *EDEM3* | ER degradation enhancer, mannosidase alpha-like 3 |
| *EFCAB7* | EF-hand calcium binding domain 7 |
| *ETV3L* | ets variant 3-like |
| *EVI5* | ecotropic viral integration site 5 |
| *EXTL2* | exostoses (multiple)-like 2 |
| *F5* | coagulation factor V (proaccelerin, labile factor) |
| *FAAH* | fatty acid amide hydrolase |
| *FAM189B* | chromosome 1 open reading frame 2 |
| *FAM63A* | family with sequence similarity 63, member A |
| *FCER1A* | Fc fragment of IgE, high affinity I, receptor for; alpha polypeptide |
| *FCRL1* | Fc receptor-like 1 |
| *FCRL2* | Fc receptor-like 2 |
| *FCRLB* | Fc receptor-like B |
| *FGGY* | FGGY carbohydrate kinase domain containing |
| *FLG* | Filaggrin |
| *FMO4* | flavin containing monooxygenase 4 |
| *GABPB2* | GA binding protein transcription factor, beta subunit 2 |
| *GBP1* | guanylate binding protein 1, interferon-inducible, 67kDa |
| *GBP6* | guanylate binding protein family, member 6 |
| *GJA5* | gap junction protein, alpha 5, 40kDa |
| *GLRX2* | glutaredoxin 2 |
| *GLT25D2* | glycosyltransferase 25 domain containing 2 |
| *GNAT2* | guanine nucleotide binding protein (G protein), alpha transducing activity polypeptide 2 |
| *GPR25* | G protein-coupled receptor 25 |
| *GPSM2* | G-protein signaling modulator 2 (AGS3-like, C. elegans) |
| *GTF2B* | general transcription factor IIB |
| *HCN3* | hyperpolarization activated cyclic nucleotide-gated potassium channel 3 |
| *HENMT1* | HFM1, ATP-dependent DNA helicase homolog (S. cerevisiae) |
| *HFM1* | human immunodeficiency virus type I enhancer binding protein 3 |
| *HIVEP3* | hemicentin 1 |
| *HMCN1* | hook homolog 1 (Drosophila) |
| *HOOK1* | hydroxy-delta-5-steroid dehydrogenase, 3 beta- and steroid delta-isomerase 1 |
| *HYI* | hydroxypyruvate isomerase homolog (E. coli) |
| *IFI16* | interferon, gamma-inducible protein 16 |
| *IGSF3* | immunoglobulin superfamily, member 3 |
| *IGSF9* | immunoglobulin superfamily, member 9 |
| *INADL* | InaD-like (Drosophila) |
| *IPO9* | importin 9 |
| *IVL* | Involucrin |
| *IVNS1ABP* | influenza virus NS1A binding protein |
| *KIAA1324* | KIAA1324 |
| *KIAA1614* | KIAA1614 |
| *KIF2C* | kinesin family member 2C |
| *KIFAP3* | kinesin-associated protein 3 |
| *LAMC1* | laminin, gamma 1 (formerly LAMB2) |
| *LAMC2* | laminin, gamma 2 |
| *LEPRE1* | leucine proline-enriched proteoglycan (leprecan) 1 |
| *LIX1L* | Lix1 homolog (mouse)-like |
| *LRRC7* | leucine rich repeat containing 7 |
| *LRRC8B* | leucine rich repeat containing 8 family, member B |
| *LRRC8C* | leucine rich repeat containing 8 family, member C |
| *MAGI3* | membrane associated guanylate kinase, WW and PDZ domain containing 3 |
| *METTL11B* | methyltransferase like 11B |
| *MIER1* | mesoderm induction early response 1 homolog (Xenopus laevis) |
| *MOBKL2C* | MOB1, Mps One Binder kinase activator-like 2C (yeast) |
| *MPL* | myeloproliferative leukemia virus oncogene |
| *MRPL37* | mitochondrial ribosomal protein L37 |
| *MSH4* | mutS homolog 4 (E. coli) |
| *NCF2* | neutrophil cytosolic factor 2 |
| *NEGR1* | neuronal growth regulator 1 |
| *NPHS2* | nephrosis 2, idiopathic, steroid-resistant (podocin) |
| *ODF2L* | outer dense fiber of sperm tails 2-like |
| *OMA1* | OMA1 homolog, zinc metallopeptidase (S. cerevisiae) |
| *OR10J1* | olfactory receptor, family 10, subfamily J, member 1 |
| *OR6P1* | olfactory receptor, family 6, subfamily P, member 1 |
| *OVGP1* | oviductal glycoprotein 1, 120kDa |
| *PAQR6* | progestin and adipoQ receptor family member VI |
| *PDE4DIP* | hypothetical protein LOC100134230; similar to KIAA0454 protein; similar to phosphodiesterase 4D interacting protein isoform 2; phosphodiesterase 4D interacting protein |
| *PEAR1* | platelet endothelial aggregation receptor 1 |
| *PIAS3* | protein inhibitor of activated STAT, 3 |
| *PIGK* | phosphatidylinositol glycan anchor biosynthesis, class K |
| *PIK3R3* | phosphoinositide-3-kinase, regulatory subunit 3 (gamma) |
| *PKLR* | pyruvate kinase, liver and RBC |
| *PODN* | Podocan |
| *POLR3C* | polymerase (RNA) III (DNA directed) polypeptide C (62kD) |
| *PPM1J* | protein phosphatase 1J (PP2C domain containing) |
| *PRPF38B* | PRP38 pre-mRNA processing factor 38 (yeast) domain containing B |
| *PRRC2C* | proline-rich coiled-coil 2C |
| *PRUNE* | prune homolog (Drosophila) |
| *RBM15* | RNA binding motif protein 15 |
| *RBMXL1* | regulatory factor X, 5 (influences HLA class II expression) |
| *RFX5* | Rh family, B glycoprotein (gene/pseudogene) |
| *RHBG* | ring finger protein 220 |
| *RTCD1* | RNA terminal phosphate cyclase domain 1 |
| *RWDD3* | RWD domain containing 3 |
| *SARS* | seryl-tRNA synthetase |
| *SEMA6C* | sema domain, transmembrane domain (TM), and cytoplasmic domain, (semaphorin) 6C |
| *SGIP1* | SH3-domain GRB2-like (endophilin) interacting protein 1 |
| *SHCBP1L* | solute carrier family 27 (fatty acid transporter), member 3 |
| *SLC27A3* | solute carrier family 44, member 5 |
| *SLC44A5* | solute carrier family 5 (sodium/glucose cotransporter), member 9 |
| *SMG7* | Smg-7 homolog, nonsense mediated mRNA decay factor (C. elegans) |
| *SPATA6* | spermatogenesis associated 6 |
| *SPRR3* | small proline-rich protein 3 |
| *SPTA1* | spectrin, alpha, erythrocytic 1 (elliptocytosis 2) |
| *TARS2* | threonyl-tRNA synthetase 2, mitochondrial (putative) |
| *TBX15* | T-box 15 |
| *TBX19* | T-box 19 |
| *TDRD5* | tudor domain containing 5 |
| *TMEM125* | transmembrane protein 125 |
| *TMEM59* | transmembrane protein 59 |
| *TMEM61* | transmembrane protein 61 |
| *TNR* | tenascin R (restrictin, janusin) |
| *TOMM40L* | translocase of outer mitochondrial membrane 40 homolog (yeast)-like |
| *TPR* | translocated promoter region (to activated MET oncogene) |
| *TRIM45* | tripartite motif-containing 45 |
| *TRIM46* | tripartite motif-containing 46 |
| *TTF2* | transcription termination factor, RNA polymerase II |
| *TXNIP* | thioredoxin interacting protein |
| *UBQLN4* | ubiquilin 4 |
| *UHMK1* | U2AF homology motif (UHM) kinase 1 |
| *UQCRH* | ubiquinol-cytochrome c reductase hinge protein-like; ubiquinol-cytochrome c reductase hinge protein |
| *UROD* | uroporphyrinogen decarboxylase |
| *VAV3* | vav 3 guanine nucleotide exchange factor |
| *VSIG8* | chromosome 1 open reading frame 204; V-set and immunoglobulin domain containing 8 |
| *WDR3* | WD repeat domain 3 |
| *ZBTB7B* | zinc finger and BTB domain containing 7B |
| *ZFYVE9* | zinc finger, FYVE domain containing 9 |
| *ZNF281* | zinc finger protein 281 |
| *ZNF687* | zinc finger protein 687 |
| *ZYG11A* | zyg-11 homolog A (C. elegans) |
| *SLC5A9* | solute carrier family 5 (sodium/sugar cotransporter), member 9 |
| *CITED4* | Cbp/p300-interacting transactivator, with Glu/Asp rich carboxy-terminal domain, 4 |
| *TNN* | tenascin N |
| *DPYD* | dihydropyrimidine dehydrogenase |
| *ASPM* | abnormal spindle microtubule assembly |
| *HSD3B1* | hydroxy-delta-5-steroid dehydrogenase, 3 beta- and steroid delta-isomerase 1 |
| *COL24A1* | collagen, type XXIV, alpha 1 |
| *RNF220* | ring finger protein 220 |
| *WDR77* | WD repeat domain 77 |

**Supplementary Table 4B: Genes in the LOD-2 SI for the chromosome 2 QT linkage peak**

| **Symbol** | **Full name** |  |
| --- | --- | --- |
| *ABCA12* | ATP-binding cassette, sub-family A (ABC1), member 12 | |
| *ABCB6* | ATP-binding cassette, sub-family B (MDR/TAP), member 6 | |
| *ACSL3* | acyl-CoA synthetase long-chain family member 3 | |
| *ADAM23* | ADAM metallopeptidase domain 23 | |
| *ALPP* | alkaline phosphatase, placental (Regan isozyme) | |
| *ANKAR* | ankyrin and armadillo repeat containing | |
| *ANKMY1* | ankyrin repeat and MYND domain containing 1 | |
| *ANKZF1* | ankyrin repeat and zinc finger domain containing 1 | |
| *AP1S3* | adaptor-related protein complex 1, sigma 3 subunit | |
| *AQP12B* | aquaporin 12B | |
| *ARMC9* | armadillo repeat containing 9 | |
| *ASNSD1* | asparagine synthetase domain containing 1 | |
| *ATF2* | activating transcription factor 2 | |
| *C2orf62* | chromosome 2 open reading frame 62 | |
| *C2orf67* | chromosome 2 open reading frame 67 | |
| *CAPN10* | calpain 10 | |
| *CASP10* | caspase 10, apoptosis-related cysteine peptidase | |
| *CCDC141* | coiled-coil domain containing 141 | |
| *CCDC150* | coiled-coil domain containing 150 | |
| *CDK15* | PFTAIRE protein kinase 2 | |
| *CNPPD1* | cyclin Pas1/PHO80 domain containing 1 | |
| *COL4A3* | collagen, type IV, alpha 3 (Goodpasture antigen) | |
| *COL5A2* | collagen, type V, alpha 2 | |
| *COL6A3* | collagen, type VI, alpha 3 | |
| *CRYGA* | crystallin, gamma A | |
| *CRYGC* | crystallin, gamma C | |
| *CYP27A1* | cytochrome P450, family 27, subfamily A, polypeptide 1 | |
| *DES* | Desmin | |
| *DGKD* | diacylglycerol kinase, delta 130kDa | |
| *DNAH7* | dynein, axonemal, heavy chain 7 | |
| *DNAJC10* | DnaJ (Hsp40) homolog, subfamily C, member 10 | |
| *ECEL1* | endothelin converting enzyme-like 1 | |
| *EFHD1* | EF-hand domain family, member D1 | |
| *ESPNL* | espin-like | |
| *FBXO36* | F-box protein 36 | |
| *FN1* | fibronectin 1 | |
| *GLB1L* | galactosidase, beta 1-like | |
| *GPR35* | G protein-coupled receptor 35 | |
| *GTF3C3* | general transcription factor IIIC, polypeptide 3, 102kDa | |
| *HDAC4* | histone deacetylase 4 | |
| *IDH1* | isocitrate dehydrogenase 1 (NADP+), soluble | |
| *IGFBP5* | insulin-like growth factor binding protein 5 | |
| *IQCA1* | IQ motif containing with AAA domain 1 | |
| *ITGAV* | integrin, alpha V (vitronectin receptor, alpha polypeptide, antigen CD51) | |
| *KIAA1486* | KIAA1486 protein | |
| *KIF1A* | kinesin family member 1ª | |
| *KLHL30* | kelch-like 30 (Drosophila) | |
| *MYO1B* | myosin IB | |
| *NDUFS1* | NADH dehydrogenase (ubiquinone) Fe-S protein 1, 75kDa (NADH-coenzyme Q reductase) | |
| *NEUROD1* | neurogenic differentiation 1 | |
| *NOP58* | NOP58 ribonucleoprotein homolog (yeast) | |
| *OBSL1* | obscurin-like 1 | |
| *PAX3* | paired box 3 | |
| *PDE11A* | phosphodiesterase 11ª | |
| *PER2* | period homolog 2 (Drosophila) | |
| *PIKFYVE* | phosphoinositide kinase, FYVE finger containing | |
| *PLEKHM3* | pleckstrin homology domain containing, family M, member 3 | |
| *SERPINE2* | serpin peptidase inhibitor, clade E (nexin, plasminogen activator inhibitor type 1), member 2 | |
| *SPAG16* | sperm associated antigen 16 | |
| *SPHKAP* | SPHK1 interactor, AKAP domain containing | |
| *SSFA2* | sperm specific antigen 2 | |
| *STK16* | serine/threonine kinase 16 | |
| *STK36* | serine/threonine kinase 36, fused homolog (Drosophila) | |
| *TMEM198* | transmembrane protein 198 | |
| *TNS1* | tensin 1 | |
| *TTN* | Titin | |
| *UNC80* | chromosome 2 open reading frame 21 | |
| *WIPF1* | WAS/WASL interacting protein family, member 1 | |
| *WNT10A* | wingless-type MMTV integration site family, member 10ª | |
| *ZFAND2B* | zinc finger, AN1-type domain 2B | |
| *ZSWIM2* | zinc finger, SWIM-type containing 2 | |

**Supplementary Table 4C: Genes in the LOD-2 SI for the chromosome 1 QRS linkage peak**

| **Symbol** | **Full name** |
| --- | --- |
| *AK2* | adenylate kinase 2 |
| *ALDH4A1* | aldehyde dehydrogenase 4 family, member A1 |
| *C1orf38* | chromosome 1 open reading frame 38 |
| *C1orf63* | chromosome 1 open reading frame 63 |
| *C1orf94* | chromosome 1 open reading frame 94 |
| *CCDC28B* | coiled-coil domain containing 28B |
| *COL16A1* | collagen, type XVI, alpha 1 |
| *DHDDS* | dehydrodolichyl diphosphate synthase |
| *E2F2* | E2F transcription factor 2 |
| *EPB41* | erythrocyte membrane protein band 4.1 (elliptocytosis 1, RH-linked) |
| *EPHA10* | EPH receptor A10 |
| *GJB4* | gap junction protein, beta 4, 30.3kDa |
| *HSPG2* | heparan sulfate proteoglycan 2 |
| *KIAA0319L* | KIAA0319-like |
| *LDLRAP1* | low density lipoprotein receptor adaptor protein 1 |
| *MACF1* | microtubule-actin crosslinking factor 1 |
| *MAN1C1* | mannosidase, alpha, class 1C, member 1 |
| *MAP3K6* | mitogen-activated protein kinase kinase kinase 6 |
| *MUL1* | mitochondrial E3 ubiquitin ligase 1 |
| *MYOM3* | myomesin family, member 3 |
| *NBPF3* | neuroblastoma breakpoint family, member 3 |
| *OTUD3* | OTU domain containing 3 |
| *PADI4* | peptidyl arginine deiminase, type IV |
| *PHACTR4* | phosphatase and actin regulator 4 |
| *PHC2* | polyhomeotic homolog 2 (Drosophila) |
| *RAP1GAP* | RAP1 GTPase activating protein |
| *RCC2* | regulator of chromosome condensation 2 |
| *RLF* | rearranged L-myc fusión |
| *SEPN1* | selenoprotein N, 1 |
| *SERINC2* | serine incorporator 2 |
| *SRRM1* | serine/arginine repetitive matrix 1 |
| *SYF2* | SYF2 homolog, RNA splicing factor (S. cerevisiae) |
| *TINAGL1* | tubulointerstitial nephritis antigen-like 1 |
| *TMCO2* | transmembrane and coiled-coil domains 2 |
| *TMCO4* | transmembrane and coiled-coil domains 4 |
| *TMEM234* | transmembrane protein 234 |
| *UBXN11* | UBX domain protein 11 |
| *USP48* | ubiquitin specific peptidase 48 |
| *ZBTB40* | zinc finger and BTB domain containing 40 |
| *GRHL3* | grainyhead-like transcription factor 3 |

**Supplementary Table 4D: Genes in the LOD-2 SI for the chromosome 9 PR linkage peak**

| **Symbol** | **Full name** |
| --- | --- |
| *GALT* | galactose-1-phosphate uridylyltransferase |
| *ALDH1B1* | aldehyde dehydrogenase 1 family, member B1 |
| *CA9* | carbonic anhydrase IX |
| *CCIN* | Calicin |
| *DDX58* | DEAD (Asp-Glu-Ala-Asp) box polypeptide 58 |
| *DENND4C* | DENN/MADD domain containing 4C |
| *FAM154A* | family with sequence similarity 154, member A |
| *FREM1* | FRAS1 related extracellular matrix 1 |
| *FRMPD1* | FERM and PDZ domain containing 1 |
| *GRHPR* | glyoxylate reductase/hydroxypyruvate reductase |
| *IFNA10* | interferon, alpha 10 |
| *IFNA14* | interferon, alpha 14 |
| *IFT74* | intraflagellar transport 74 homolog (Chlamydomonas) |
| *KIAA1045* | KIAA1045 |
| *KIAA1797* | KIAA1797 |
| *NOL6* | nucleolar protein family 6 (RNA-associated) |
| *OR2S2* | olfactory receptor, family 2, subfamily S, member 2 |
| *PLIN2* | adipose differentiation-related protein |
| *PTPLAD2* | protein tyrosine phosphatase-like A domain containing 2 |
| *RECK* | reversion-inducing-cysteine-rich protein with kazal motifs |
| *RGP1* | RGP1 retrograde golgi transport homolog (S. cerevisiae) |
| *UBAP1* | ubiquitin associated protein 1 |
| *UNC13B* | unc-13 homolog B (C. elegans) |

**Supplementary Table 4E: Genes in the LOD-2 SI for the chromosome 14 PR linkage peak**

| **Original name** | **Full name** |
| --- | --- |
| *RNASE4* | ribonuclease, RNase A family, 4 |
| *ADCY4* | adenylate cyclase 4 |
| *ARHGEF40* | Rho guanine nucleotide exchange factor 40 |
| *CEBPE* | CCAAT/enhancer binding protein (C/EBP), epsilon |
| *CMA1* | chymase 1, mast cell |
| *HEATR5A* | HEAT repeat containing 5A |
| *KLHL33* | kelch-like 33 (Drosophila) |
| *LRP10* | low density lipoprotein receptor-related protein 10 |
| *LRRC16B* | leucine rich repeat containing 16B |
| *MMP14* | matrix metallopeptidase 14 (membrane-inserted) |
| *MYH6* | myosin, heavy chain 6, cardiac muscle, alpha |
| *OR10G3* | olfactory receptor, family 10, subfamily G, member 3 |
| *OR4K1* | olfactory receptor, family 4, subfamily K, member 1 |
| *OR4N5* | olfactory receptor, family 4, subfamily N, member 5 |
| *PARP2* | poly (ADP-ribose) polymerase 2 |
| *PCK2* | phosphoenolpyruvate carboxykinase 2 (mitochondrial) |
| *PRKD1* | protein kinase D1 |
| *PSMB11* | proteasome (prosome, macropain) subunit, beta type, 11 |
| *RABGGTA* | Rab geranylgeranyltransferase, alpha subunit |
| *RBM23* | RNA binding motif protein 23 |
| *SDR39U1* | short chain dehydrogenase/reductase family 39U, member 1 |
| *SLC7A7* | solute carrier family 7 (cationic amino acid transporter, y+ system), member 7 |
| *SUPT16H* | suppressor of Ty 16 homolog (S. cerevisiae); suppressor of Ty 16 homolog (S. cerevisiae) pseudogene |
| *TEP1* | telomerase-associated protein 1 |
| *TTC5* | tetratricopeptide repeat domain 5 |
