# Supplementary material for: A Combined Linkage and Exome Sequencing Analysis for Electrocardiogram Parameters in the Erasmus Rucphen Family Study
Source: Front Genet. 2016 Nov 8;7:190. doi: 10.3389/fgene.2016.00190 (PMC5099142; doi:10.3389/fgene.2016.00190)
Supplement: Supplementary file 2 [file Data_Sheet_2.docx]

**Supplementary Figure 1. Genes Networks for selected damaging genes associated with candidate genes FCRL2, TTN, SPHKAP, DMRTA2, MPL and CEP350**

**A.**


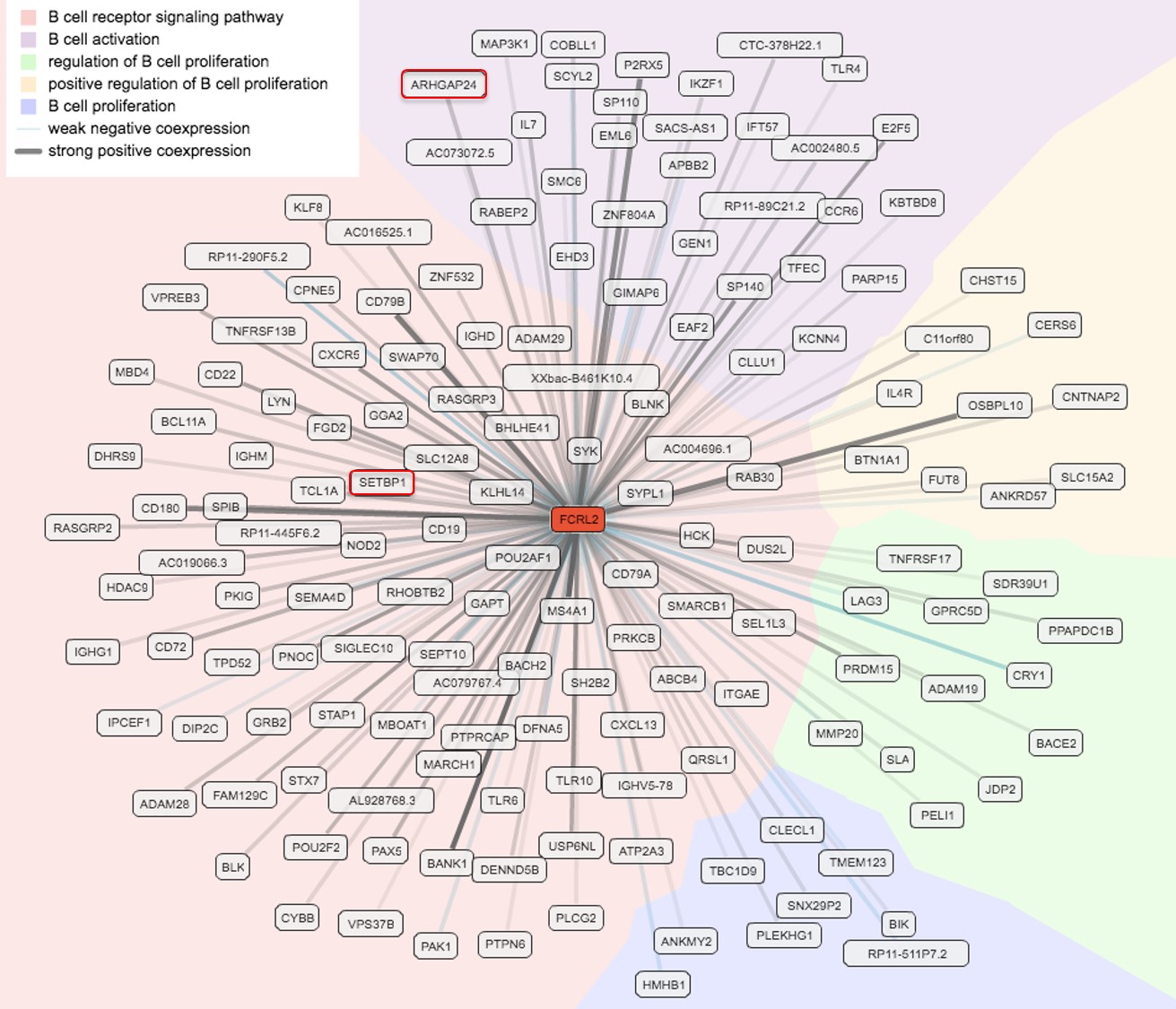


Among 157 coexpressed genes, FCRL2 interacts with *ARHGAP24* (PR associated gene), *SETBP1* (QRS associated gene)

**B.**

**
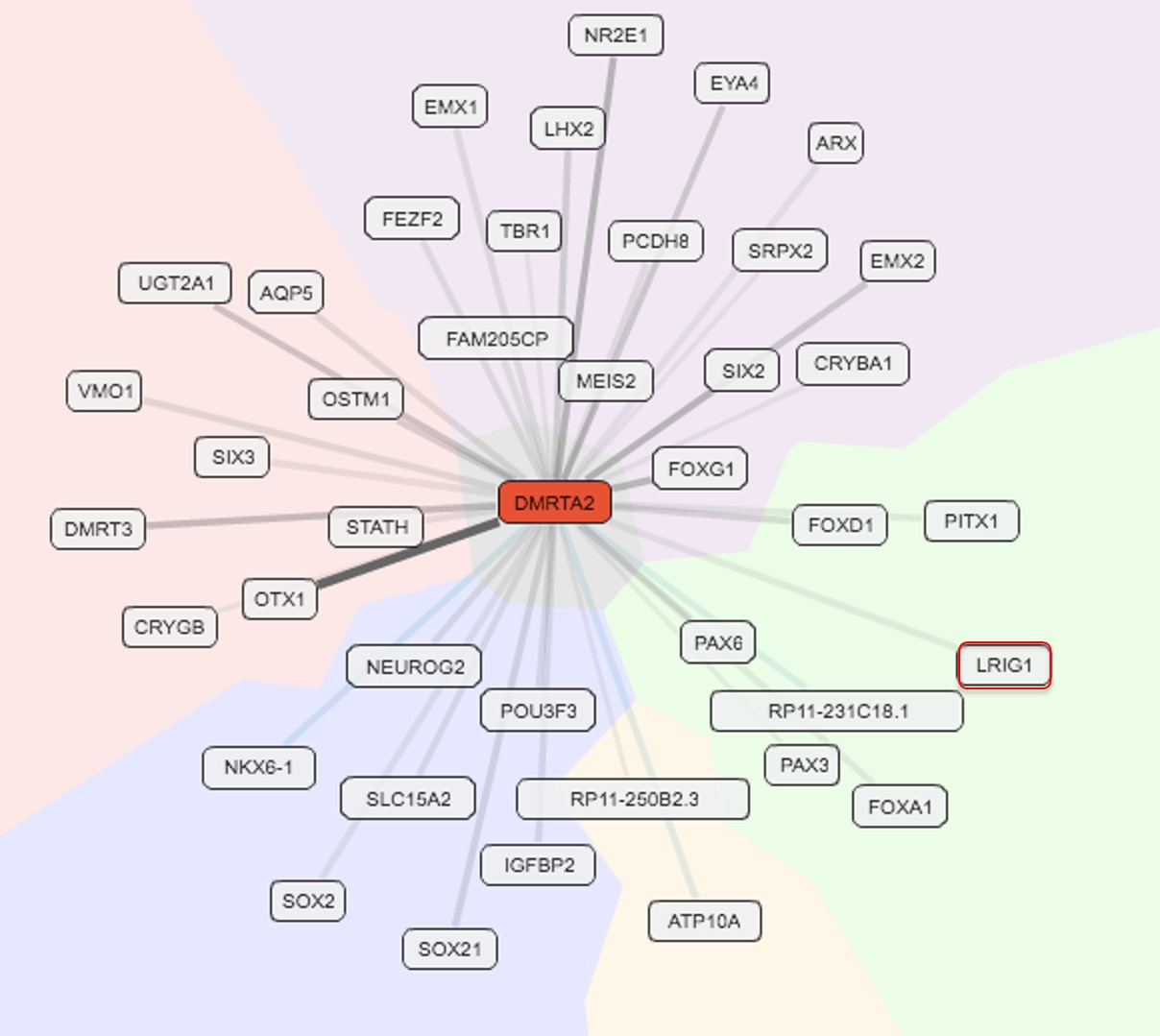
**

*DMRTA2* is coexpressed with 41 genes; among these, *LRIG1*, a QRS gene, emerged

**C.**

**
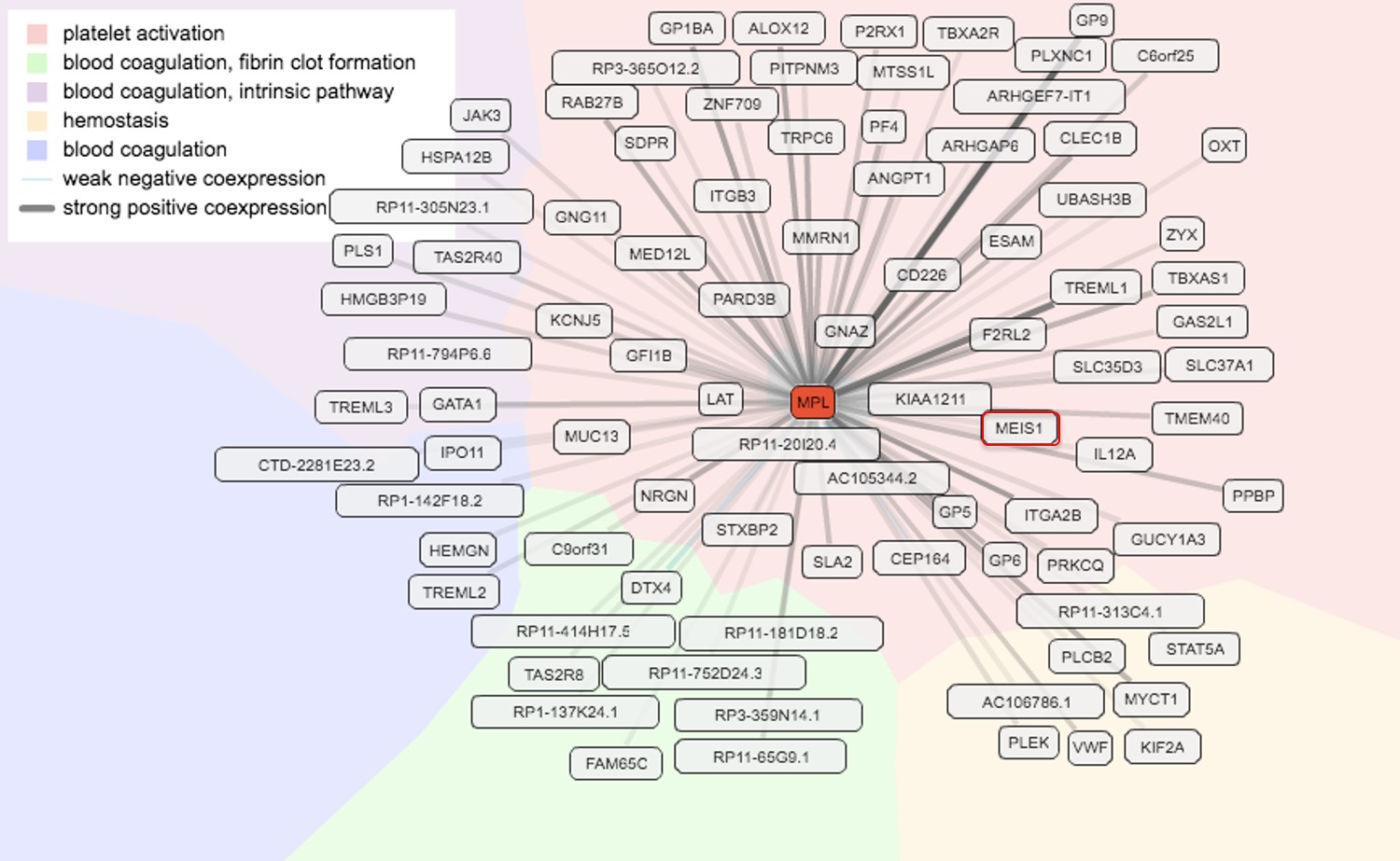
**

*MPL* is coexpressed with 89 genes, including *MEIS1* a PR gene

**D.**


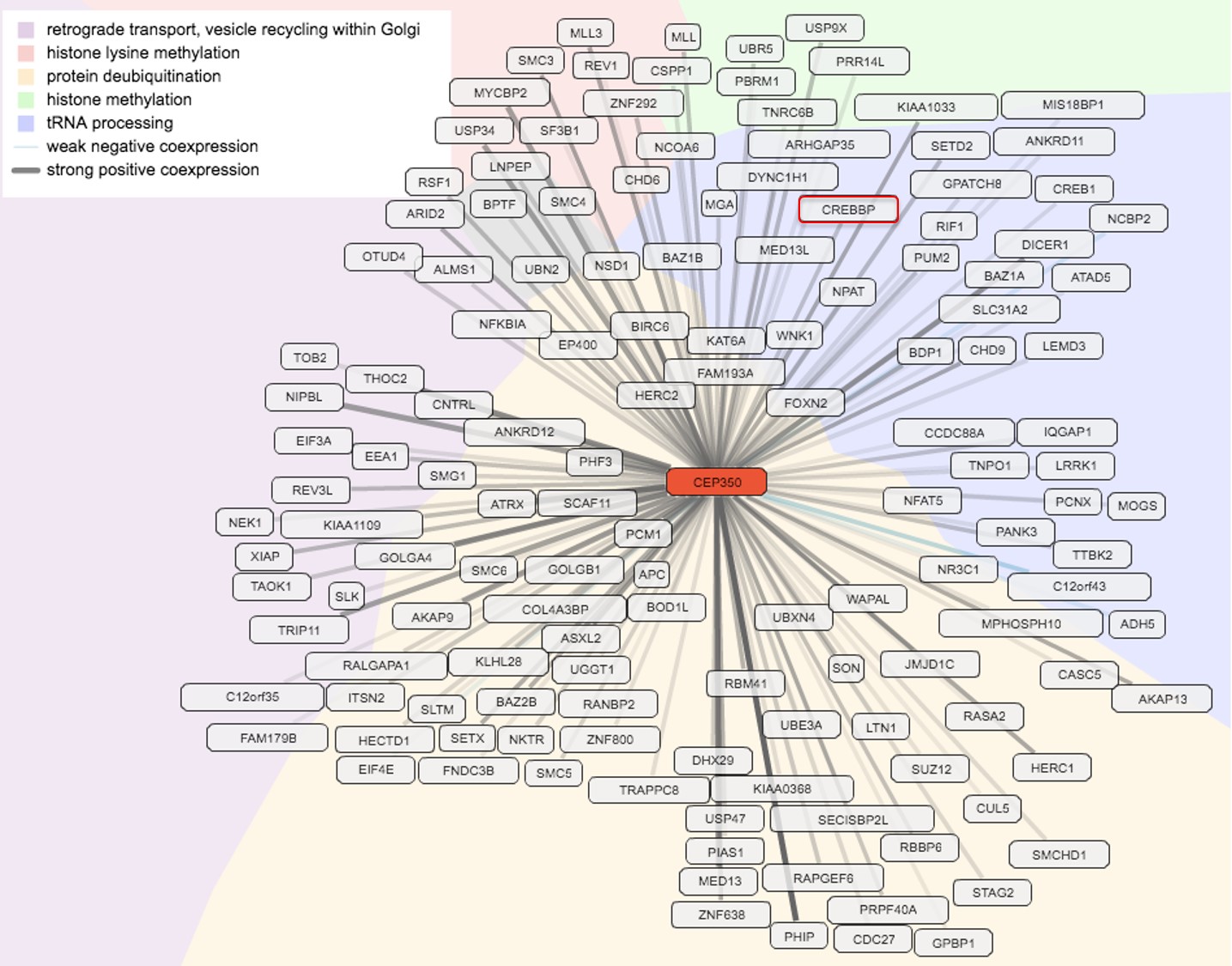


The coexpression network for *CEP350* has 142 genes, among these, CREBBP a candidate gene for QT

**E.**

**
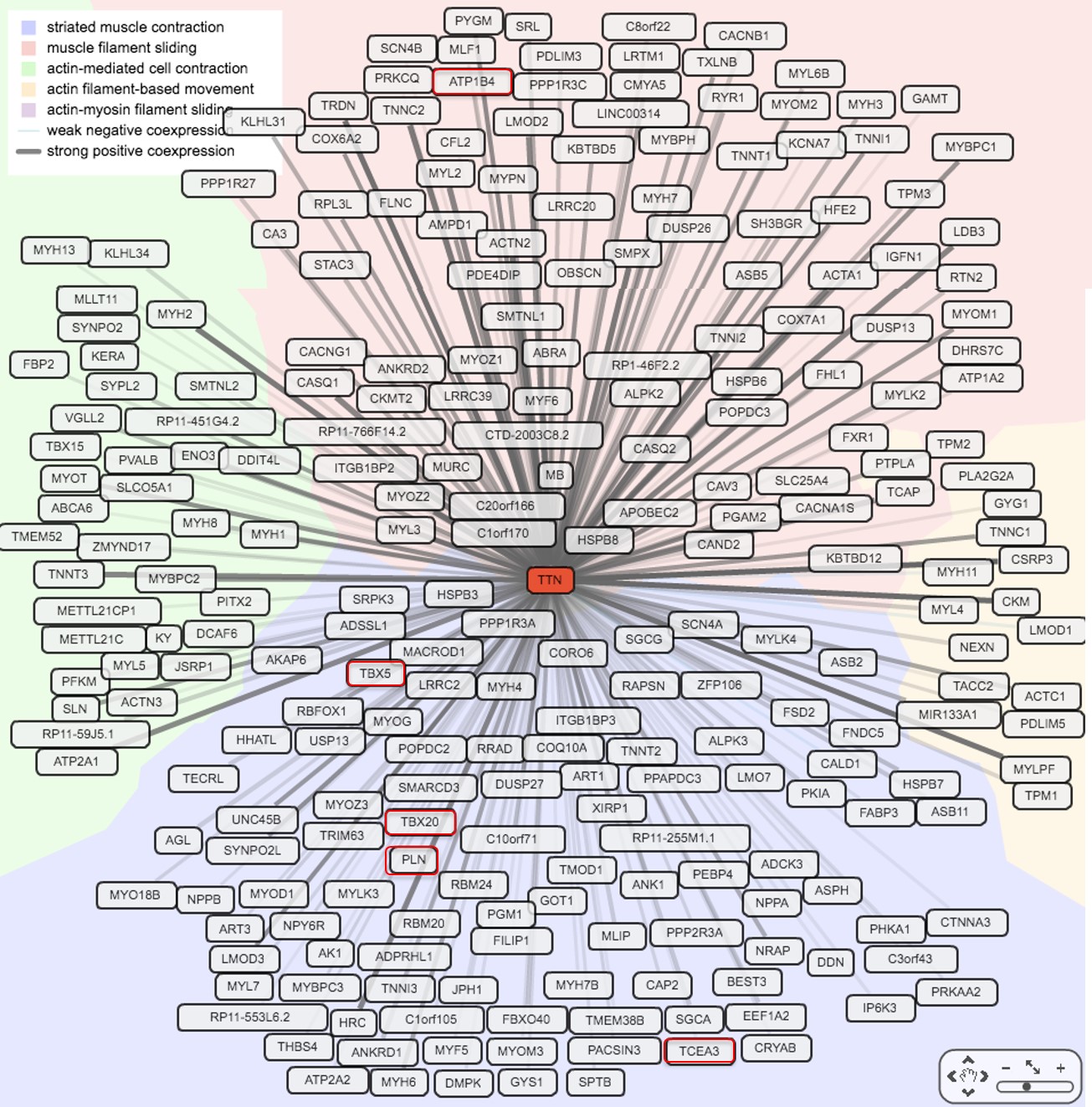
**

Among 253 coexpressed genes, QT gene *TTN* interacts with five others including QT interval associated genes (*TCEA3*, *PLN, ATP1B)* and QRS interval genes (*TBX20*, *TBX5)*

**F.**

**
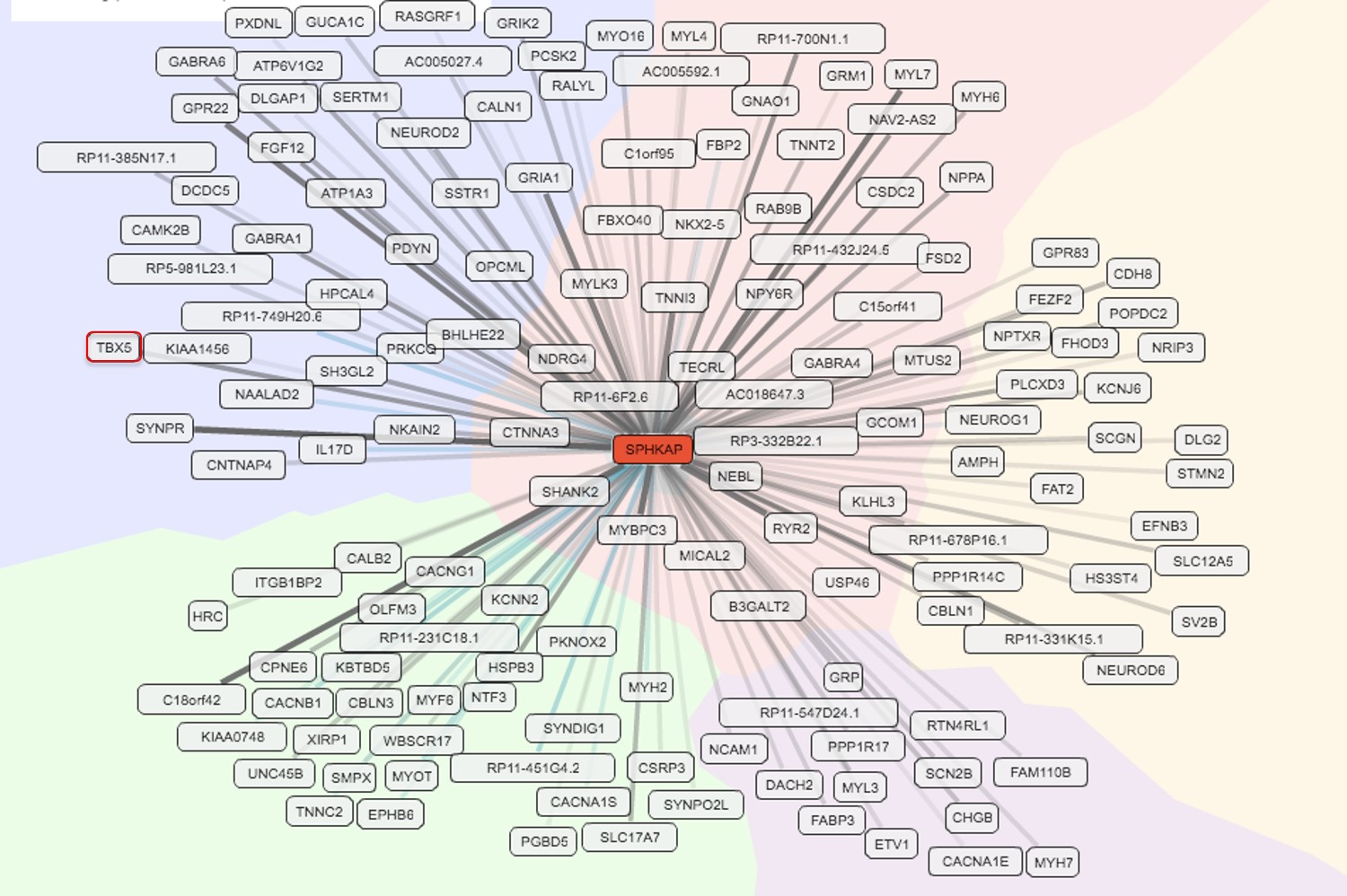
**

*SPHKAP* is part of a coexpression module containing 148 genes, *TBX5,* a QRS and PR candidate gene, among them
